# Therapeutic Inertia in the New Landscape of Multiple Sclerosis Care

**DOI:** 10.3389/fneur.2018.00174

**Published:** 2018-03-20

**Authors:** Gustavo Saposnik, Xavier Montalban

**Affiliations:** ^1^Outcomes and Decision Neuroscience lab, Division of Neurology, Department of Medicine, St. Michael’s Hospital, University of Toronto, Toronto, ON, Canada; ^2^Laboratory for Social and Neural Systems Research, Department of Economics, University of Zurich, Zurich, Switzerland; ^3^Neurology-Neuroimmunology Department, Neurorehabilitation Unit, Multiple Sclerosis Centre of Catalonia (Cemcat), Barcelona, Spain; ^4^Center for Multiple Sclerosis, St. Michael’s Hospital, University of Toronto, Toronto, ON, Canada

**Keywords:** therapeutic inertia, multiple sclerosis, decision-making, humanized antibodies, outcomes

## Abstract

The landscape of multiple sclerosis (MS) treatment is constantly changing. Significant heterogeneity exists in the efficacy and risks associated with these therapies. Therefore, clinicians have the challenge to tailor treatment based on several factors (disease activity level, risk of progression, individual patient preferences and characteristics, personal expertise, etc.), to identify the optimal balance between safety and efficacy. However, most clinicians have limited education in decision-making and formal training in risk management. Together, these factors may lead to therapeutic inertia (TI); defined as the absence of treatment initiation or intensification when therapeutic goals are unmet. TI may lead to suboptimal treatments choices, worse clinical outcomes, and more disability. This article provides a succinct overview on factors influencing TI in MS care.

The reason men oppose progress is not that they hate progress, but that they love inertia—Elbert Hubbard (writer 1859–1915)

The landscape of multiple sclerosis (MS) has changed over the last few years. Clinicians and patients welcomed the introduction of disease-modifying therapy (DMT) for MS in the mid-1990s. Injectable agents, all with rather similar risk–benefit profiles, dominated MS care for over a decade (1). The approval of Natalizumab—a recombinant monoclonal antibody that reduces signs of disease activity and inflammation—for MS treatment marked a paradigm change with the introduction of a more effective treatment option, but also the realization of the risks associated with modulation of the immune system (e.g., risk of PML) (2). More recently, the introduction of oral agents has opened yet another avenue for patients and clinicians. Currently, there are over 12 DMTs available to treat MS, with varying availability around the world. Significant heterogeneity exists in the efficacy and risks associated with these therapies. Therefore, clinicians have the challenge to tailor treatment based on (i) disease activity level, (ii) risk of progression, (iii) individual patient preferences and characteristics, and (iv) personal expertise, to identify the optimal balance between safety and efficacy. Based on the aforementioned factors, neurologists caring for MS patients face important choices in each medical encounter: (1) continue with the same management, (2) initiate or escalate therapy for a more effective or safer agent, or (3) consider a reassessment within months under the uncertainty of the current status of the patient. As a result, therapeutic inertia (TI)—first coined by Okonofua (3) for management of patients with hypertension and diabetes—emerged to define the absence of treatment initiation or intensification in patients when therapeutic goals are unmet. Physician factors (e.g., low tolerance to uncertainty, *status quo* bias) are considered to be the main contributors to TI (explaining at least 50% of TI) but remain poorly studied (Table 1) (4). One of the invoked explanations is physicians’ limited training in risk management and formal learning in medical decision-making. Furthermore, patients, commonly less informed about therapeutic options (e.g., efficacy and risk of side effects of DMTs), have limited tools to participate in a shared decision. This situation may lead to suboptimal treatments choices, worse clinical outcomes, and more disability (5).

**Table 1:** Factors influencing therapeutic inertia in multiple sclerosis (MS) care.^a^

Physicians factors	Patient-related factors	Health-care factors
Failure to set clear goals	Demographic (e.g., older age)	Lack of guidelines
Errors in risk assessment	Misinterpretation of clinical activity (e.g., non-disabling attacks)	Coverage and funding for disease-modifying therapies (DMTs) (government, HMOs, etc.)
Failure to identify comorbid conditions influencing clinical outcomes	Radiological activity	Lack of visit planning
Underestimation of patient’s need	Aversion to change	Lack of contingency plans for patients experiencing new symptoms
Low tolerance to uncertainty	Concomitant mental illness (e.g., depression affecting self-care)	Limited resources (e.g., MS clinic space, busy schedules, low clinic, and MRI capacity)
Aversion to unknown risks/*status quo*	Side effects of new DMTs	High costs
Herding (mistakenly following a colleague previous decision)	Poor communication	Lack of coordination of health-care services
Nihilistic approach	Lack of trust	
Knowledge gaps (lack of awareness of clinical guidelines)		

*^a^Adapted from Reach et al. (5) and Cooke et al. (4) with focus in MS care*.

Previous studies in MS care revealed that a more proactive management (e.g., including earlier use of high-efficacy DMTs and close monitoring of the clinical and radiological response to treatment) may slow the disease progression, disability, cognitive impairment, and MRI activity (6–9).

To tackle treatment inertia in MS, we might apply concepts from neuroeconomics, the science that studies the principles of how we make decisions. For example, classic studies in consumer research showed that the higher the number of available options may negatively influence consumer’s decisions due to information overload (10). Moreover, the time of the day influences assertive decisions, phenomenon called “decision fatigue.” Similarly, 7 out of 10 neurologists expressed TI as a result of these factors and lower tolerance to uncertainty (11). This is not surprising given that neurology practice is a medical specialty with higher incidence of physicians’ biases and burnout, and MS being a neurological condition leading the paradigm of multiple therapeutic choices with ongoing developments—altogether the perfect combination for TI (https://www.medscape.com/slideshow/lifestyle-2016-overview-6007335, accessed December 15, 2017).

This finding was also observed in previous meta-analysis including physicians (12). The authors found at least one bias in 50% of physicians. Most common identified biases include: overconfidence, lower tolerance to uncertainty, the anchoring effect, information, and availability bias (13).

Some educational strategies were developed to overcome physicians’ biases. An experimental study in Rotterdam tested the benefits of reflective reasoning to diagnostic accuracy in 36 medical residents (14). Reflective reasoning is a strategy that incorporates the analysis of case scenario by identifying findings that were present or expected, and those that support or were against the diagnosis. The authors found significant improvement among second-year residents (2.03; 95% CI, 1.49–2.57) and the first-year residents (2.31; 95% CI, 1.89–2.73) exposed to the intervention (14). A pilot study in MS care, applied the traffic light system (TLS) as an educational intervention that facilitates the decision-making process. The TLS emerged as a warning and risk categorization strategy to reduce human errors by facilitating the integration of specific situations with an action (15–18). In MS care, the goal was to match case scenarios with three types of situations according to the risk of progression: red light (“high risk”/“stop and think”), yellow light (warning, reassessment is needed), and green light (“stable”/“continue the same strategy”). The authors found this educational intervention was feasible and promising by showing a trend toward a reduction in TI compared with controls (OR 0.57; 95% CI 0.26–1.22) (11).

Another key factor in patients’ therapeutic choices relates to the application of the prospect theory (developed by Dr. Amos Tversky and Dr. Daniel Kahneman in 1979 that merit winning the Novel prize of Economy in 2002) (19). The prospect theory describes the way people choose between alternatives that involve risk, where the probabilities of outcomes are known. For example, individuals may choose $50 for sure over the 50% probability of winning $100 (same utility) depending on the reference point (i.e., a sure $50 may represent a more meaningful winning to someone with a low income compared with someone wealthier). The prospect theory helps understand people choices based on their risk preferences and the reference point. This theory can be applied to MS treatment: patients with a low risk of progression or non-disabling attacked may be less willing to choose more risky (less safer) treatments, whereas those with high risk of progression may be willing to take an effective treatment that carries on a higher risk of complications (Figure 1). The EMPOWER study showed that patients’ preferences in DMT selection were mainly driven by minimizing risks of side effects, the route of administration, and treatment schedule (20).

**Figure 1:**
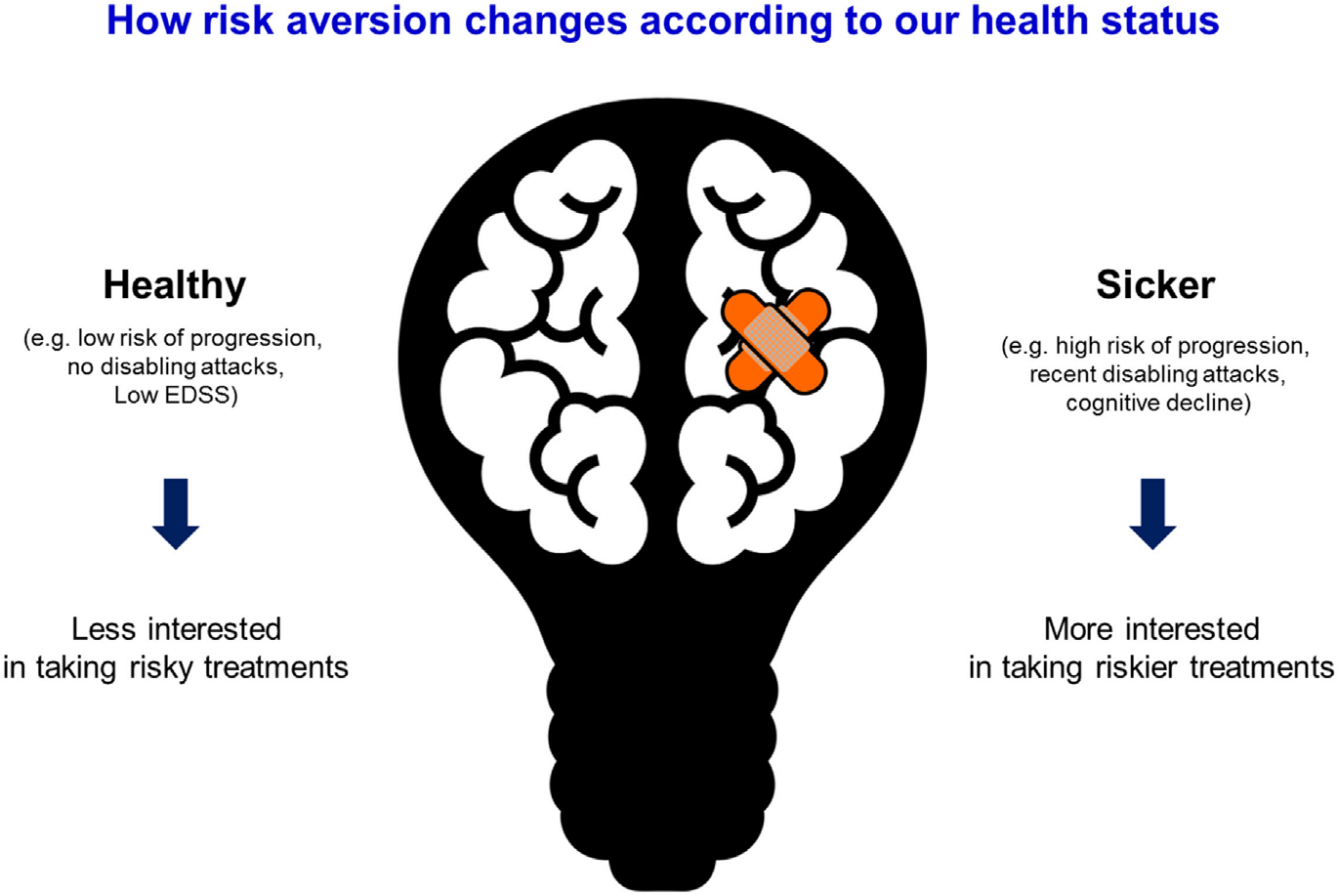
Application of the prospect theory to multiple sclerosis care.

How about the treating physician? Are we ready to escalate therapy for high-risk patients when treatment goals are unmet? Studies evaluating the management of hypertension, diabetes, atrial fibrillation, and MS suggest that 50–70% of clinicians do not escalate therapy when indicated by best practice guidelines (3, 21).

Finally, a third important concept is the human tendency to the *status quo* (tendency to maintain previous choices) and default bias (keep the option preselected by others). Common examples include individuals’ tendency to keep the same insurance, phone, TV cable, or Internet provider despite other more valuable options are available. Patients and health-care providers are not immune to those biases and may miss an opportunity for improvement. On the other hand, this phenomenon may explain the appropriate resistance to escalate therapies in MS patients under uncertainty (e.g., controversial situations, unclear evidence of clinical relapses) or insurance barriers for medication switches when not clearly justified.

New treatments represent new opportunities to control MS. We, as clinicians involved in MS care, need to be aware of our own biases. More comprehensive studies that evaluate the efficacy of educational interventions to ameliorate medical errors and suboptimal therapeutic choices are needed.

Medical Schools and Scientific Institutions should be involved given the lead role in facilitating a medical education: formal training in risk management and decision-making.

Our role as caring physicians is to provide information to our patients to facilitate therapeutic decisions. Meanwhile, our patients may have to assume a more active role in the decision-making process given the new paradigm in MS treatment. This broad spectrum of therapeutic choices should lead to a shared over unilateral decisions. Paraphrasing Nelson Mandela (Political leader and Philanthropist; 1918–2013): “*Education is the most powerful weapon which you can use to change the world*.” We need to educate ourselves to guide and coach our patients toward a more informed decision process.

## Author Contributions

GS: study concept and design, data interpretation, manuscript drafting, and creation of tables and figures. XM: study concept, data interpretation, and supervision.

## Conflict of Interest Statement

The authors declare that the research was conducted in the absence of any commercial or financial relationships that could be construed as a potential conflict of interest.
